# Nanomedicine and therapy of lung diseases

**DOI:** 10.1590/S1679-45082014MD3113

**Published:** 2014

**Authors:** Fabrício de Melo Garcia

**Affiliations:** 1 Faculdade de Medicina Nova Esperança, João Pessoa, PB, Brazil.

**Keywords:** Nanomedicine, Drug delivery systems, Nanotechnology, Lung diseases/therapy

## Abstract

The use of nanotechnology has significantly increased in different fields of science, including the development of drug delivery systems. Currently, the most modern pharmaceutical nanocarriers, such as liposomes, micelles, nanoemulsions and polymeric nanoparticles, demonstrate extremely useful properties from the point of view of drug therapy. In this context, the development of nanocarriers for pulmonary application has been much debated by the scientific community in recent decades. Although research on the use of nanoparticles for pulmonary application are still in the initial phase, the studies conducted to date suggest that the development of drug delivery systems for systemic or local treatment of diseases that affect the respiratory system may be promising.

## INTRODUCTION

Nanomedicine is related to developing, characterizing, and applying treatment systems on a nanometric or micrometric scale.^(1,2)^ Studies of such systems have been carried out at several research centers worldwide, aimed at directing and controlling the release of drugs.^(3,4)^

Historically, the employment of most drugs has been limited by several pharmacokinetic factors. Among them, the impossibility of increasing their concentration in the blood, the amount of time the therapeutic agent remains in the circulation, the low solubility and, especially the undesirable side effects inherent to high-dose treatments. These factors make it difficult to attain the necessary concentration of the drug for pharmacological and therapeutic success.^(3,5)^

Currently, thanks to more recent studies in nanomedicine, the biological properties of nanoparticles can be changed and controlled. This is enabled by changes on the surface of these particles.^(1,2)^ More modern pharmaceutical nanocarriers, such as liposomes, micelles, nanoemulsions and polymeric nanoparticles showed extremely useful properties from the pharmacological and therapeutic standpoint.^(6)^

Among these properties, the increased circulation time of the drug in the blood, allowing the accumulation in pathological areas with compromised or inflamed vascularization, and the increase in specificity as to site of action, in addition to better penetration in the tissues affected stand out.^(2,6)^

The development of nanocarriers for the lungs has been very much debated by the scientific community in the past decades.^(7) ^ In general, carrier systems comprised by nanoparticles are an attractive concept for using pharmaceuticals that are active on the respiratory system. This occurs due to possible increased retention of these particles in lung tissue along with increase in drug release time, mainly when used in large porous matrices of nanoparticles.^(5)^

Another interesting fact is that some studies demonstrated that the absorption of nanoparticles by macrophages may be reduced if the particles are smaller than 260nm.^(5)^ These combined effects (increased retention and non-uptake by the phagocyte system) may improve local treatment of drugs with pulmonary action.^(5)^

Thus, the present article aimed to briefly discuss some of the major advantages of developing new nanotechnological structures used in lung disease treatment, underscoring some of the most recent studies in the area.

## Development of nanoparticles used in lung therapy

Studies on the lung administration route is a field of growing interest, not only for local treatment of respiratory tract conditions, but also for the systemic administration of drugs, especially in the case of lipophilic ones, that have low bioavailability when given per oris.^(7)^

Inhaled medications are convenient and extremely efficacious in airway treatment, allowing treatment with a high degree of specificity and retention of a high concentration of the drug in the target-tissue. This generates low systemic exposure to the drug and reduction of systemic side effects.^(8)^

The interest in solid lipid nanoparticles and in lipid nanostructured carriers and other nanocarriers (lipossomes, nanoemulsions and polymeric nanoparticles) as a therapeutic alternative for pulmonary application has grown in past years. However, research on the development of lipid nanoparticles that can be administered per pulmonary route is still in an initial phase.^(7)^

Using airways has some advantages as compared to oral and injectable administration. In addition to avoiding first-pass metabolism, reducing secondary effects and the therapeutic dose of the drug,^(7)^ the pulmonary administration allows local release of therapeutic agents which is an important differential in the treatment of respiratory diseases, such as asthma, chronic obstructive pulmonary disease (COPD) and cystic fibrosis.^(9)^

The large lung surface area (over 100m^2^) and the thin epithelial layer that covers the airways (0.2 to 1μm width), added to ample vascularization, allow fast absorption of pulmonary route administered drugs.^(7,9)^ Moreover, the relatively low enzyme activity helps keep bioavailability of drugs high in this route, and the non-invasive nature of the pulmonary route increases patient compliance to treatment.^(9)^

Some pharmacokinetic and pharmacodynamic properties of nanoparticles may be the differential in the treatment of lung disease, among which: (1) late or controlled absorption of the drug; (2) restricted biodistribution; (3) increased retention time of drug at site of action; (4) increased specificity of the drug to affected tissue; and (5) reduced side effects and toxicity of the drug.^(3)^ These properties are attained and modulated by using technologies that allow handling the structure of the different types of nanoparticles, in addition to using different materials in the composition of these pharmaceutical formulations.^(3)^

Currently, several inhaled nanopharmaceuticals are being developed. Among drugs undergoing tests, budesonide, salbutamol, itraconazole and paclitaxel can be mentioned.^(9)^

Several studies with aerosol formulations of solid nanoparticles containing glucocorticoids demonstrated a significant improvement in the pharmacokinetics of these drugs. Such studies may lead to the development of new pharmaceutical formulations containing pulmonary route administrable corticosteroids.^(7)^

Some liposomal antibacterials targeted at pulmonary infections are already in phase II of clinical trials.^(8)^ Among them, amikacin and ciprofloxacin. These studies showed that several treatment cycles with liposomal amikacin have to sustainable improvement in pulmonary function, and a significant reduction in bacterial density in patients with cystic fibrosis associated with chronic pulmonary infections caused by *Pseudomonas sp*.^(8)^ These studies suggest increased efficacy of these antibacterial agents when administered in the liposomal format.

Another area of major interest involves studies on new formulations with pharmaceuticals for the treatment of tuberculosis. Studies with nanoparticles with three pharmaceuticals (isoniazid, pyrazinamide and rifampin), already known for the treatment of tuberculosis, were performed on guinea pigs and demonstrated significant increase in bioavailability and in the circulation time of these drugs in the body of the animals studied.^(7,10)^ These studies suggest that using nanoparticles as carriers of tuberculostatic drugs administered by the pulmonary route may be promising in future studies.

The toxicity of these nanoparticles used as pulmonary route drug carriers has also been studied. Nanoparticles used by the pulmonary route seem to have a low toxicological potential, although continued studies are needed to check for possible long-term toxicity, taking into account the repeated use of these new coming medications.^(7)^

Although studies on nanoparticles for pulmonary application are still in an initial phase, studies performed so far suggest that nanoparticles are an interesting option in the systemic or local treatment of respiratory diseases.
