# Supplementary material for: Nanomedicine and therapy of lung diseases
Source: Einstein (Sao Paulo). 2014 Oct-Dec;12(4):531–3. doi: 10.1590/S1679-45082014MD3113 (PMC4879928; doi:10.1590/S1679-45082014MD3113)
Supplement: Translation

# Translation: Nanomedicina e terapia de doenças pulmonares

**Authors:** Fabrício de Melo Garcia

**Affiliations:** 1 Faculdade de Medicina Nova Esperança, João Pessoa, PB, Brasil.

**Keywords:** Nanomedicina, Sistemas de liberação de medicamentos, Nanotecnologia, Pneumopatias/terapia

## Abstract

O uso da nanotecnologia tem aumentado significativamente em diversas áreas da ciência. Entre elas, está o desenvolvimento de sistemas de liberação de medicamentos. Atualmente, os nanocarreadores farmacêuticos mais modernos, como os lipossomas, as micelas, as nanoemulsões e as nanopartículas poliméricas, demonstram propriedades extremamente úteis do ponto de vista farmacoterápico. Nesse contexto, o desenvolvimento de nanocarreadores para aplicação pulmonar tem sido um tema amplamente debatido pela comunidade científica nas últimas décadas. Embora as pesquisas sobre o uso de nanopartículas para aplicação pulmonar ainda estejam em fase inicial, estudos realizados até hoje sugerem que o delineamento de sistemas de liberação de medicamentos para o tratamento sistêmico ou local de doenças que afetam o sistema respiratório, pode ser promissor no desenvolvimento de novas terapias de doenças pulmonares.

## INTRODUÇÃO

A nanomedicina está relacionada ao desenvolvimento, à caracterização e à aplicação de sistemas terapêuticos em escala nanométrica ou micrométrica.^(1,2)^ Estudos de tais sistemas têm sido realizados em diversos centros de pesquisa em todo mundo, com o propósito de direcionar e controlar a liberação de fármacos.^(3,4)^

Ao longo da história, a utilização da maioria dos fármacos foi limitada por diversos fatores farmacocinéticos. Entre eles, a impossibilidade do aumento da sua concentração no sangue, o tempo de permanência do agente terapêutico na circulação, a baixa solubilidade e, especialmente, os efeitos colaterais indesejáveis inerentes às terapias com doses elevadas. Esses fatores dificultam a utilização da concentração necessária do fármaco para o sucesso farmacoterapêutico.^(3,5)^

Atualmente, graças aos estudos mais recentes na área da nanomedicina é possível modificar e controlar as propriedades biológicas das nanopartículas. Isso ocorre por meio de alterações da superfície dessas estruturas.^(1,2)^ Os nanocarreadores farmacêuticos mais modernos, tais como os lipossomas, as micelas, as nanoemulsões e as nanopartículas poliméricas, demonstram propriedades extremamente úteis do ponto de vista farmacoterápico.^(6)^

Entre essas propriedades, pode-se destacar o aumento do tempo de circulação do fármaco no sangue, permitindo o acúmulo em áreas patológicas com vascularização comprometida ou inflamadas, e o aumento da especificidade quanto ao local da ação, além de uma melhor penetração nos tecidos afetados.^(2,6)^

O desenvolvimento de nanocarreadores para aplicação pulmonar tem sido um tema amplamente debatido pela comunidade científica nas últimas décadas.^(7) ^Em geral, o uso de sistemas carreadores compostos por nanopartículas é conceitualmente atraente para utilização de fármacos que atuam no sistema respiratório. Isso ocorre devido ao possível aumento de retenção dessas partículas no tecido pulmonar acompanhado da elevação do tempo de liberação do fármaco, principalmente quando são utilizadas grandes matrizes porosas de nanopartículas.^(5)^

Outro fato interessante, é que alguns estudos demonstraram que a absorção das nanopartículas por macrófagos pode ser reduzida se as partículas forem menores que 260nm.^(5)^ Esses efeitos combinados (aumento da retenção e a não captura pelo sistema fagocitário) podem melhorar a terapia local de fármacos com ação pulmonar.^(5)^

Dessa forma, este artigo visou discutir sucintamente algumas das principais vantagens do desenvolvimento de novas formas nanotecnológicas utilizadas nas terapias de doenças pulmonares, destacando alguns dos estudos mais recentes nessa área.

### Desenvolvimento de nanopartículas utilizadas na terapia pulmonar

Estudos sobre a via de administração pulmonar é um campo de interesse crescente, não só para o tratamento local de doenças das vias respiratórias, mas também para a administração sistêmica de medicamentos, especialmente no caso de fármacos lipofílicos, que apresentam uma baixa biodisponibilidade quando administrados pela via oral.^(7)^

Medicamentos inalantes são convenientes e extremamente eficazes no tratamento das vias aéreas, permitindo uma terapia com alto grau de especificidade e retenção de altas concentrações do fármaco no tecido-alvo. Isso gera uma baixa exposição sistêmica à droga e redução dos efeitos colaterais sistêmicos.^(8)^

O interesse nas nanopartículas lipídicas sólidas e nos carreadores nanoestruturados lipídicos, bem como outros nanocarreadores (lipossomas, nanoemulsões e nanopartículas poliméricas), como alternativa terapêutica para aplicação pulmonar, aumentou nos últimos anos. No entanto, as investigações acerca do desenvolvimento de nanopartículas lipídicas administráveis pela via pulmonar ainda estão em fase inicial.^(7)^

A administração pela via pulmonar tem algumas vantagens em relação a outras vias, como a oral e a injetável. Além de evitar o metabolismo de primeira passagem, reduzindo os efeitos secundários e a dose terapêutica do fármaco,^(7)^ a administração pulmonar permite a liberação local dos agentes terapêuticos, sendo um diferencial importante no tratamento de doenças respiratórias, como a asma, a doença pulmonar obstrutiva crônica (DPOC) e a fibrose cística.^(9)^

A grande área de superfície pulmonar (mais do que 100m^2^) e a fina camada epitelial que recobre as vias aéreas (0,2 a 1μm de espessura), somadas a ampla vascularização, permitem uma absorção rápida de fármacos administrados pela via pulmonar.^(7,9)^ Além disso, a relativa baixa atividade enzimática ajuda a manter a alta biodisponibilidade dos fármacos nessa via, e a natureza não invasiva da via pulmonar aumenta a adesão do paciente ao tratamento.^(9)^

Algumas propriedades farmacocinéticas e farmacodinâmicas das nanopartículas podem fazer o diferencial no tratamento de doenças pulmonares, entre elas: (1) absorção retardada ou controlada do fármaco; (2) biodistribuição restrita; (3) aumento do tempo de retenção do fármaco no local da ação; (4) aumento da especificidade do fármaco ao tecido afetado; e (5) redução dos efeitos colaterais e toxicidade do fármaco.^(3)^ Essas propriedades são obtidas e moduladas a partir do uso de tecnologias que permitem a manipulação da estrutura dos diferentes tipos de nanopartículas, além do uso de diferentes materiais na composição dessas formas farmacêuticas.^(3)^

Atualmente, diversos nanofármacos inalantes estão sendo desenvolvidos. Entre os fármacos que ainda estão passando por testes, podem-se citar a budesonida, o salbutamol, o itraconazol e o paclitaxel.^(9)^

Diversas pesquisas com formulações de nanopartículas sólidas em aerossol, contendo glicocorticoides demonstraram uma melhora significativa da farmacocinética desses fármacos. Tais estudos podem levar ao desenvolvimento de novas formas farmacêuticas contendo corticosteroides administráveis pela via pulmonar.^(7)^

Tendo como alvo as infecções pulmonares, alguns antibacterianos lipossomais já estão na fase II de ensaios clínicos.^(8)^ Entre eles, a amicacina e a ciprofloxacina. Esses estudos mostraram que vários ciclos de tratamento com amicacina lipossomal levaram a melhora sustentada da função pulmonar, além da redução significativa da densidade bacteriana em pacientes com fibrose cística associada a infecções pulmonares crônicas causadas por *Pseudomonas sp*.^(8)^ Essas pesquisas sugerem o aumento da eficácia desses antibacterianos quando administrados na forma lipossomal.

Outra área de grande interesse envolve as pesquisas acerca de novas formulações contendo fármacos para o tratamento da tuberculose. Estudos com nanopartículas contendo três fármacos (isoniazida, pirazinamida e rifampicina), já conhecidos para o tratamento da tuberculose foram realizados em cobaias, esses ensaios demonstraram significativo aumento da biodisponibilidade e do tempo de circulação desses fármacos no organismo dos animais estudados.^(7,10)^ Essas pesquisas sugerem que o uso de nanopartículas como carreadoras de drogas tuberculostáticas administradas pela via pulmonar pode ser promissor em estudos posteriores.

A toxicidade dessas nanopartículas utilizadas como carreadores de fármacos pela via pulmonar também tem sido alvo de estudos. As nanopartículas utilizadas pela via pulmonar parecem apresentar baixo potencial toxicológico, entretanto são necessário estudos continuados para verificar uma possível toxicidade a longo prazo, isto levando em consideração o uso repetitivo desses novos medicamentos que estão por vir.^(7)^

Embora as pesquisas sobre o uso de nanopartículas para aplicação pulmonar ainda estejam em fase inicial, as pesquisas realizadas até o momento sugerem que as nanopartículas são uma opção interessante no tratamento sistêmico ou local de doenças que afetam o sistema respiratório.
